# Oxygen isotope in archaeological bioapatites from India: Implications to climate change and decline of Bronze Age Harappan civilization

**DOI:** 10.1038/srep26555

**Published:** 2016-05-25

**Authors:** Anindya Sarkar, Arati Deshpande Mukherjee, M. K. Bera, B. Das, Navin Juyal, P. Morthekai, R. D. Deshpande, V. S. Shinde, L. S. Rao

**Affiliations:** 1 Department of Geology and Geophysics, Indian Institute of Technology, Kharagpur 721302, India; 2 Deccan College Post Graduate and Research Instiute, Pune 411006, India; 3 Physical Research Laboratory Navrangpura, Ahmedabad 380009, India; 4 Birbal Sahni Institute of Palaeosciences, Lucknow, India; 5 Archaeological Survey of India, Nagpur, 440006, India

## Abstract

The antiquity and decline of the Bronze Age Harappan civilization in the Indus-Ghaggar-Hakra river valleys is an enigma in archaeology. Weakening of the monsoon after ~5 ka BP (and droughts throughout the Asia) is a strong contender for the Harappan collapse, although controversy exists about the synchroneity of climate change and collapse of civilization. One reason for this controversy is lack of a continuous record of cultural levels and palaeomonsoon change in close proximity. We report a high resolution oxygen isotope (δ^18^O) record of animal teeth-bone phosphates from an archaeological trench itself at Bhirrana, NW India, preserving all cultural levels of this civilization. Bhirrana was part of a high concentration of settlements along the dried up mythical Vedic river valley ‘Saraswati’, an extension of Ghaggar river in the Thar desert. Isotope and archaeological data suggest that the pre-Harappans started inhabiting this area along the mighty Ghaggar-Hakra rivers fed by intensified monsoon from 9 to 7 ka BP. The monsoon monotonically declined after 7 ka yet the settlements continued to survive from early to mature Harappan time. Our study suggests that other cause like change in subsistence strategy by shifting crop patterns rather than climate change was responsible for Harappan collapse.

The rise of the post-Neolithic Bronze Age Harappan civilization 5.7–3.3 ka BP (ca. 2500 to 1900 year BC; all ages henceforth mentioned are in cal year BP) spread along the Indus Valley of Pakistan through the plains of NW India, including into the state of Gujarat and up to the Arabian Sea and its decline has remained an enigma in archaeological investigation^1^,^2^,^3^,^4^,^5^,^6^. In the Indian subcontinent the major centers of this civilization include Harappa and Mohenjo-Daro in Pakistan and Lothal, Dholavira and Kalibangan in India (Fig. 1A). In recent years excavation at Rakhigarhi and few other places indicate that the civilization probably was more expansive than thought before^7^,^8^,^9^. Whatever may be the extent most Harappan settlements grew in the floodplains of river systems including those of the Indus or now defunct Ghaggar-Hakra (mythical river Saraswati?). Climatically although these regions fall under the influence of the Indian summer monsoon, they are currently semi-arid receiving much lesser rainfall than the mainland India. Because the monsoon showed significant variation over, both on short and long term time scale, throughout the Holocene period, attempts have been made to relate the evolution of the Harappan civilization to the changes in monsoon. Accordingly, the flourishing Harappan civilization and its decline have been linked to the intensification of monsoon during the Mid-Holocene climate optimum and its subsequent weakening, respectively. The evidence comes from a variety of sources like distant lake sediments in the Thar desert^10^,^11^, foraminiferal oxygen isotopes in Arabian sea cores^12^, fluvial morphodynamics^3^, and climate models^13^. Although the collapse of the Harappan as well as several contemporary civilisations like Akkadian (Mesopotamia), Minoan (Crete), Yangtze (China) has been attributed to either weakening of monsoon or pan-Asian aridification (drought events) at ~4.1 ka^6^,^10^,^11^, the evidence is both contradictory and incomplete. Either the climatic events and cultural levels are asynchronous^11^,^14^,^15^ or the climate change events themselves are regionally diachronous^16^ and references therein).

Potential reasons for these conflicting interpretations is that the climate reconstructions were made from locations (e.g., Thar Desert or Arabian Sea) distant from the main Harappan settlement areas or that the climate proxies (e.g., sedimentology and geochemistry in lakes) could have been influenced by multiple local parameters apart from mere rainfall or temperature. To date no continuous climate record has existed close to or from the Harappan settlements. Here we report a high resolution bulk oxygen isotope (δ^18^O) record of animal teeth and bone phosphates (bioapatites) from an excavated archaeological trench at Bhirrana, state of Haryana, NW India, to reconstruct a paleomonsoonal history of the settlement site itself. Based on radiocarbon ages from different trenches and levels the settlement at Bhirrana has been inferred to be the oldest (>9 ka BP) in the Indian sub-continent^8^,^17^,^18^. To check its validity we dated archaeological pottery from two cultural levels using optically stimulated luminescence (OSL) method and thus investigated the interrelationship between the cultural levels and climate change that occurred right at the settlement, a critical gap in information that exists in our present understanding of the Harappan civilization.

## Harappan civilization and archaeology of Bhirrana

**Figure 1:**
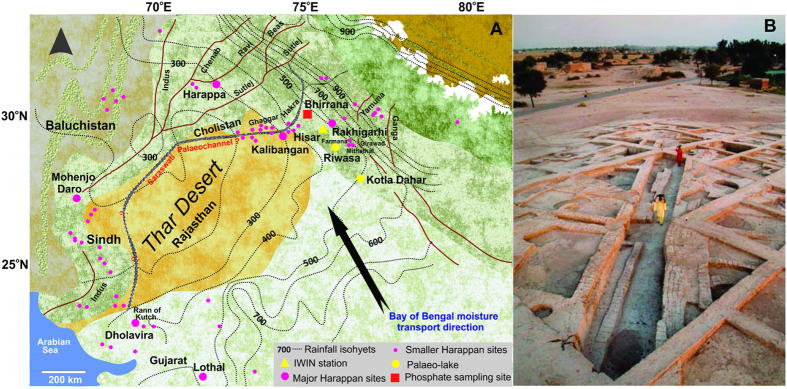
(**A**) Map of Northwest India and Pakistan (created by Coreldraw x7; http://www.coreldraw.com) showing the locations of main Harappan settlements including phosphate sampling site of Bhirrana, Haryana, IWIN precipitation sampling station at Hisar and two paleo-lakes Riwasa and Kotla Dahar studied earlier (see Fig. 3 and text for details). Black dotted lines represent 100 mm rainfall isohyets. Approximate trace of dried paleo-channel of ‘Saraswati’ (dashed white lines in Fig. 1A) is also shown. Black arrow indicates the direction of monsoon moisture transport from Bay of Bengal. (For interpretation of the references to color in this figure legend, the reader is referred to the web version of this article). Figure created by CorelDRAW Graphics Suite X7 (http://www.coreldraw.com) (**B**) Panoramic view of the excavation of mature Harappan stage at Bhirrana view from North-east (photograph reproduced with the permission of Archeological Survey of India).

Archaeological chronologies of Harappan (Indus) civilization in South Asia^2^,^16^,^19^ are given in SI. Conventionally the Harappan cultural levels have been classified into 1) an Early Ravi Phase (~5.7–4.8 ka BP), 2) Transitional Kot Diji phase (~4.8–4.6 ka BP), 3) Mature phase (~4.6–3.9 ka BP) and 4) Late declining (painted Grey Ware) phase (3.9–3.3 ka BP^13^,^19^,^20^). This chronology is based on more than 100 ^14^C dates from the site of Harappa and nearby localities. These periodization is temporally correlatable with the Indus valley civilisations from Baluchistan and Helmand province proposed by Shaffer^21^. While the first two phases were represented by pastoral and early village farming communities, the mature Harappan settlements were highly urbanized with several organized cities, developed material and craft culture having trans-Asiatic trading to regions as distant as Arabia and Mesopotamia. The late Harappan phase witnessed large scale deurbanization, population decrease, abandonment of many established settlements, lack of basic amenities, interpersonal violence and disappearance of Harappan script^22^,^23^,^24^. Although referred to as a ‘collapse’ of Harappan civilization, evidences rather suggest that smaller settlements continued albeit dispersed from original river valleys of Indus and Ghaggar-Hakra (Fig. 1A) to more distant areas of the Himalayan foothills and Ganga-Yamuna interfluves or Gujarat and Rajasthan^25^,^26^,^27^.

Based on the spatio-temporal distribution of the archaeological remains spread throughout the subcontinent a much older chronology has, however, been advocated by Possehl^2^2,^16^. Accordingly the time spans of the above four phases have been suggested as ~9–6.3 ka BP, 6.3–5.2 ka BP, 5.2–3 ka BP and 3–2.5 ka BP respectively. Clearly the later time scale pushes back the Harappan chronology to at least 1–2 ka older. Evidences of a post-Neolithic-Pre Harappan (often referred to as the Hakra ware) phase were first reported by Mughal^28^,^29^ in the Cholistan region east of the Indus valley along the Indo-Pakistan border, but have now been found from several localities in India. The Hakra settlements, spread along the Ghaggar-Hakra river valleys have been found at Kalibangan, Farmana, Girawad, Rakhigarhi and Bhirrana, the present site of investigation (Fig. 1A 30,31,32,33). A large number (~70) of conventional and AMS radiocarbon dates indeed support the antiquity of this phase in different parts of the Indus-Ghaggar Hakra river belts viz. Girawad (Pit-23, 6.2 ka BP), Mithathal (Trench A-1, 8.2 ka BP), Kalibangan (sample TF-439, 7.6 ka BP). The recent excavations at Rakhigarhi suggest hitherto unknown largest Harappan settlement in India preserving all the cultural levels including the Hakra phase (sample S-4173, 6.4 ka BP^8^,^9^,^34^,^35^).

**Figure 2:**
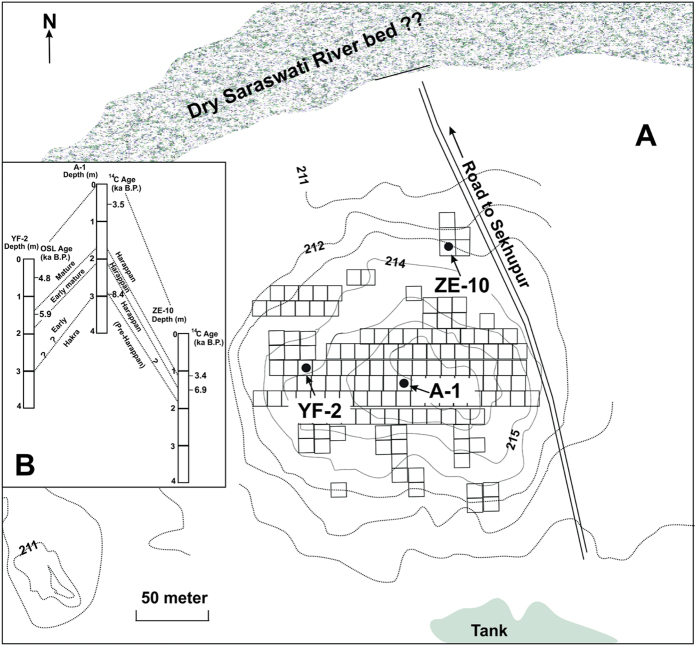
(**A**) Settlement pattern of period 1A (pre-Harappan Hakra) along with locations of trenches at Bhirrana mound. Figure created by CorelDRAW Graphics Suite X7 (http://www.coreldraw.com) (**B**) Tentative lateral time correlation of different cultural levels between the trenches based on radiocarbon and OSL dates. Contours are in cm. above msl. Only the trench YF-2 yielded continuous bioapatite samples (see text).

**Figure 3:**
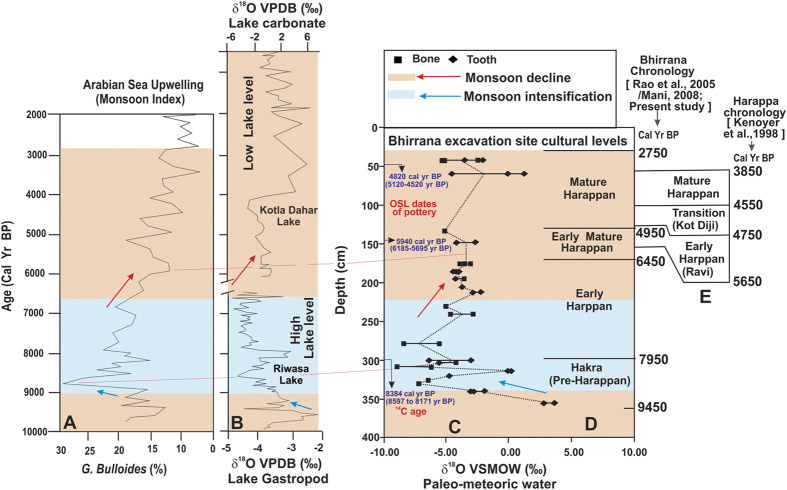
(**A**) Arabian Sea upwelling intensity as monsoon index^57^. (**B**) Carbonate δ^18^O and lake level records from paleo-lakes Riwasa and Kotla Dahar, Haryana (refs 5 and 6). (**C**) Bioapatite based paleo-meteoric water δ^18^O (monsoon proxy) record at Bhirrana along with characteristic archaeological and faunal elements from different cultural levels. Note monsoon intensification from ~9 ka to 7 ka BP (blue shaded region and arrows) and monotonous decline from ~7 ka to 2.8 ka BP (brown shaded region, red arrows); dotted pink lines denote approximate time correlation of these two phases across the sites. (**D**) Bhirrana chronology based on archaeological evidences^17^,^18^,^32^, ^14^C and new OSL dates. OSL dates are from trench YF-2; the oldest ^14^C date is from correlatable level of trench ZE-10 (**E**) Conventional chronology^19^,^20^; note new dates, archaeological evidences and climate pattern are all suggestive of a much older age for the beginning of Harappan civilization at Bhirrana.

A compilation of calibrated radiocarbon dates of the charcoal samples and OSL dates of pottery (see later discussion) from various cultural levels of Bhirrana (Lat. 29°33′N; Long. 75°33′E), retrieved during the excavation of 2005, is given in SI^8^,^18^. At Bhirrana the earliest level has provided mean ^14^C age of 8.35 ± 0.14 ka BP (8597 to 8171 years BP^8^). The successive cultural levels at Bhirrana, as deciphered from archeological artefacts along with these ^14^C ages, are Pre-Harappan Hakra phase (~9.5–8 ka BP), Early Harappan (~8–6.5 ka BP), Early mature Harappan (~6.5–5 ka BP) and mature Harappan (~5–2.8 ka BP^8^,^17^,^18^,^20^,^34^). Cultural stratigraphy of Bhirrana settlement depicting the periods, cultural levels, ages based on calibrated radiocarbon ages in different trenches and characteristic archeological artefacts and attributes are given in SI^8^,^17^,^20^. A panoramic view of the excavation of the mature Harappan level at Bhirrana view from north-east is shown in Fig. 1B. Figure 2A shows the settlement pattern of pre-Harappan Hakra phase (period 1A 8) along with locations of three major trenches at Bhirrana mound YF-2, A-1, and ZE-10. A schematic E-W cross section of the trench YF-2 depicting the cultural levels at Bhirrana is shown in SI. Fig. 2B (inset) shows the tentative lateral time correlation based on radiocarbon and OSL dates generated during present investigation (see later discussion). The Bhirrana settlement, close to the presently dried up Ghaggar-Hakra (Saraswati) river bed preserves all the major laterally traceable and time correlatable cultural levels. As expected in trench A-1, the central part of the archaeological mound, the Hakra or other phases are much thicker (>3 m) compared to the flanking trenches of YF-2 and ZE-10. At Bhirrana the Hakra ware culture period is the earliest and occurs as an independent stratigraphic horizon^17^,^34^. The Hakra phase was primarily identified by ceramics such as mud appliqué ware, incised ware, and bi-chrome ware, much similar to the Pre-Harappan phase in Cholistan (Figs 1A and 3C 36) and was characterized by its subterranean dwelling, sacrificial and industrial pits^8^,^17^,^34^. The Early Harappan phase shows settlement expansion, mud brick houses with advanced material culture including arrow heads, rings and bangles of copper; beads of carnelian, jasper, and shell; bull figurines; chert blades; terracotta bangles, etc. (Fig. 3C) 17,32,34). The early mature to mature Harappan phases yielded ceramics with geometric, floral and faunal motifs; steatite bull seals; beads of semi-precious stone, shell and terracotta; animal figurines; bangles of faience and shell; copper bangles, chisels, rings, rods, etc.^17^,^34^. The excavations also yielded large quantities of faunal remains comprising bones, teeth, horn cores, etc. from all the four periods at Bhirrana and were identified at species levels^37^. Detail methods of faunal analysis for materials from the Bhirrana trench YF2 are given in the SI. Preliminary faunal investigations suggest presence of domestic cattle e.g., cow/ox (*Bos indicus),* buffalo *(Bubalus bubalis*), goat (*Capra hircus*) and sheep (*Ovis aries*) from the earliest levels. Besides the dietary use of cattle and goats, wild fauna such as nilgai (*Boselaphas tragocamelus*), Indian spotted deer (*Axis axis*) and antelope (*Antilope cervicapra*) were also a part of the diet^37^,^38^,^39^,^40^. Representative photographs of the artefacts and animal remains from various cultural levels of Bhirrana are shown in SI.

For retrieving information on past climatic changes we isotopically analysed bulk (see SI text) teeth and bone phosphates, wherever available, from the trench YF-2 which has both stratigraphic and sampling continuity (SI Table 2). To check the validity of the radiocarbon dates and the antiquity of the Bhirrana settlement we dated two pottery fragments (SI Fig. 1) in the same trench by OSL technique from both early mature and mature Harappan intervals. Detail methodology is given in SI text. The pottery at 42 cm, identified as mature Harappan level yielded mean 4.8 ± 0.3 (1σ) ka BP age (range 5120 to 4520 year BP) while the pottery from deeper level corresponding to early mature Harappan at 143 cm yielded 5.9 ± 0.25 (1σ) ka BP age (range 6185 to 5695 year BP). Within the experimental errors both the stratigraphically controlled new ages agree with the time scale based on archaeological evidences (as well as ^14^C ages) proposed by earlier workers^8^,^17^,^18^,^34^; Fig. 3C,D) and suggest that the Bhirrana settlements are the oldest of known sites in the Ghaggar-Hakra tract. Figure 3D,E show the comparison between the conventional chronology of the Harappan civilization with the proposed chronology at Bhirrana. Clearly the Bhirrana levels are few thousand years older. The 5.9 ka age at 143 cm along with the 8.38 ka age of the Hakra level below suggest that the base of the Bhirrana section, representing initiation of Harappan settlements (Hakra phase), is older than 8 ka BP. Below we show that isotope based paleoclimatic information also lends supports to the antiquity of Harappan settlements at Bhirrana.

## Oxygen isotope (δ^18^O) in bioapatites and past monsoon record at Bhirrana excavation site

δ^18^O [defined as δ (%) = {(R_sample_ − R_reference_)/R_reference_} × 1000; R = ^18^O/^16^O ratio] composition of fossil bone or tooth enamel bioapatite [carbonated hydroxyapatite^41^] is a robust tool for estimating the past meteoric water composition (drinking water for land animals^41^,^42^,^43^,^44^,^45^,^46^) compared to carbonates which are prone to diagenetic alteration. Near-continuous teeth and bone samples were available only in trench YF-2 and have been analysed. SI Fig. 4 shows the representative teeth and bone samples analysed from all the four cultural levels of Bhirrana. The samples comprise a large variety of bioapatites from mandibular and maxillary molar teeth of cattle, goat, deer and antelope to rib and vertebra bones. Since diagenetic alteration can alter isotopic signals we investigated the animal bones under electron microprobe that suggests preservation of original bioapatites suitable for isotopic analysis (see diagenetic investigation of bioapatites in SI). Detail methods of δ^18^O analysis of bioapatites are given in SI text. Under a constant body temperature of ~37 °C, the δ^18^O in mammalian phosphate (δ^18^O_p_) essentially depends on the δ^18^O value of water (δ^18^O_w_) ingested by the organism. Between the water and phosphate, oxygen isotope is fractionated in two steps, i.e., between environmental and body water and between body water and phosphate in teeth and bones^47^,^48^. Large numbers of studies have been made on modern mammalian phosphates to constrain the interrelationship between δ^18^O_p_ and δ^18^O_w_^41^,^49^,^50^,^51^. Although in general most large mammals have been found to preserve equilibrium isotopic signature, species specific fractionation equations have also been proposed by several workers (*ibid*). For the Bhirrana mammals we used the taxon specific herbivorous mammal equations of Bryant and Froelich^47^. Because these equations are dependent on body mass it is desirable to infer paleoclimate from large body sized mammals. All Bhirrana mammals satisfy this criterion representing only cattle, deer or goats. δ^18^O_p_ data of bioapatites and calculated δ^18^O_W_ are given in Table 1 of SI.

Figure 3C shows δ^18^O_W_ variation as a function of depth and against Harappan chronology at Bhirrana proposed by Rao *et al.*^17^ and Mani^18^. In general the bulk bioapatite δ^18^O in large mammals reflects the integrated mean annual δ^18^O of local meteoric water ingested by the animal during its life time. At several cultural levels we analysed multiple samples of either teeth or both teeth and bones. The spread in estimated δ^18^O_W_ ranges from <1‰ to maximum ~4‰ and are probably due to the seasonal variation in δ^18^O_W_^52^,^53^,^54^,^55^,^56^. Because our purpose was to retrieve the mean meteoric water δ^18^O_W_ value from successive layers, we sampled bulk enamel or phosphate along the entire length of a single tooth or a bone (see SI text), yet the inter-sample seasonal signature might have been preserved in some cases. In spite of the inter-sample spread, the mean δ^18^O_W_ values (dotted line in Fig. 3C) through the levels show a clear trend. At the base of the trench section (355 cm), equivalent to ~9 ka Pre-Harappan Hakra level, the δ^18^O_W_ values are enriched (+3.75‰). The δ^18^O_W_ values rapidly decreases towards the early Harappan phase reaching δ^18^O minimum of −9.01‰ at ~8 ka (trench depth ~308 cm). Thereafter the δ^18^O_W_ monotonically gets enriched from early Harappan through early mature Harappan to mature Harappan, a time span from ~8 ka BP to 2.8 ka BP. We interpret this δ^18^O_W_ variation through all the cultural levels at Bhirrana as major change in monsoonal precipitation during the last 9.5 ka. We compare the Bhirrana record with available monsoon records from Arabian Sea (*G. bulloides* upwelling index; Fig. 3A 57) and composite gastropod-carbonate δ^18^O records from two inland lakes Riwasa and Kotla Dahar, proximal to Bhirrana (Fig. 3B; re-plotted from supplementary information in refs 5 and 6). A weak monsoon phase is identified before 9 ka BP (lower part of Hakra phase). The well constrained monsoon intensification phase from 9 ka BP to 7 ka BP (late Hakra to middle part of early Harappan) is clearly discernible in all three records (blue shaded bars in Fig. 3A–C). Monsoon monotonically declined from 7 ka BP to 2 ka BP, i.e., during later part of the early Harappan to mature Harappan phase (brown shaded bar) with concomitant lowering of lake levels (Fig. 3B). The early Holocene monsoon intensification and its subsequent decline, as recorded in Bhirrana archaeological bioapatites, have been widely documented in Asia and were principally driven by boreal summer insolation^5^,^54^,^56^. Presence of aeolian sands in lake Riwasa, higher salinity in Bay of Bengal, lower *G. bulloides* upwelling intensity and enriched δ^18^O in Arabian speleothems suggest a weak monsoon phase before 10 ka BP throughout the Asia^5^,^55^,^56^,^57^,^58^,^59^,^60^. Correspondingly the 9–7 ka monsoon intensification phase is recorded in high lake levels (negative δ^18^O), lower oceanic salinity, increased upwelling, reduction in δ^18^O in speleothems from Arabia to Tibet, higher erosion rate in the Himalayas, and increased sedimentation in the Ganges deltaic plains (*ibid*^61^,^62^,^63^,^64^,^65^,^66^). The late Holocene (7 ka onwards) gradual reduction in monsoon is also amply evident throughout the Asia.

**Figure 4:**
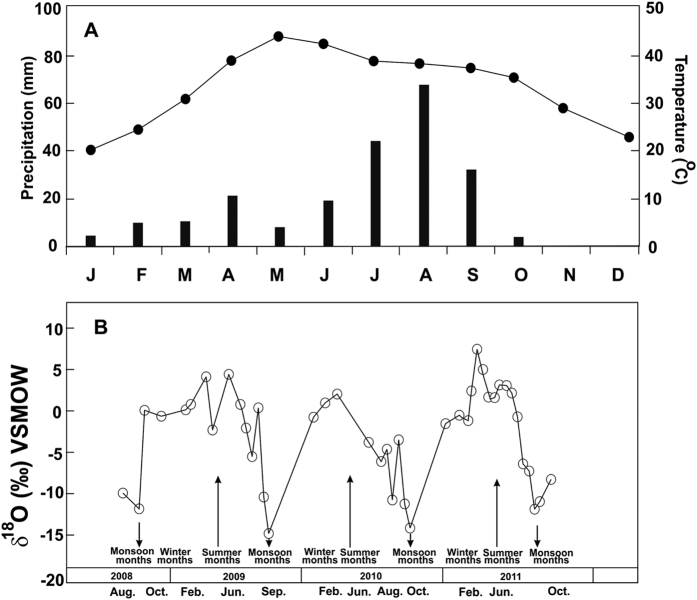
(**A**) Seasonal variation in temperature and rainfall and (**B**) Time series of precipitation δ^18^O at IWIN station Hisar, close to Bhirrana archaeological site.

Although compared to marine or lake archives the time resolution of the archaeological bioapatite based monsoon record is poor, preservation of the major phases of Holocene monsoon change combined with the OSL dates of potteries lend strong support to the antiquity of the Bhirrana settlement. To further constrain the change in paleo-meteoric water composition we generated time series δ^18^O of modern precipitation for successive three years at Hisar, a place 50 km SE of Bhirrana (Fig. 4) under the national program of ‘Isotopic Fingerprinting of Water in India (IWIN)’. As in other places of north-western India, rainfall is highest during the summer months from June to September (Fig. 4A). The monsoon moisture originates in Bay of Bengal and successively rains inland towards north-western India (Fig. 1A). The continental effect thus causes depletion in precipitation δ^18^O from −5.4‰ near the coast to −6.5‰ in north western India^67^. The modern mean annual rainfall isohyets for this part of semi-arid NW India (Fig. 1A) show that all the Harappan settlement areas (including Bhirrana) receive 400 to 600 mm precipitation compared to >1000 mm in eastern and southern India^67^. At Hisar the modern precipitation δ^18^O ranges from ~+5‰ in non-monsoon (extreme evaporation) to −15‰ in peak monsoon periods (depletion) with weighted mean annual δ^18^O value of −7‰. The large monsoon depletion in δ^18^O results from well-known amount effect where excess rainfall is known to produce extreme depletion (an increase in 100 mm of rainfall associated with a decrease in δ^18^O by 1.5‰ 67,68). The most depleted paleo-meteoric water value at Bhirrana is −9.01‰ (SI Table 2; Fig. 3C). Considering the δ^18^O_W_ value at each level represents mean annual precipitation and using a simple moisture flux model^67^, we estimate that the early Holocene (9–7 ka) monsoon precipitation at Bhirrana was ~100–150 mm higher than today. The subsequent enrichment from 7 ka onwards (by more than 6‰) reaching maximum towards the mature Harappan time indicates very low rainfall generating mean annual δ^18^O_W_ similar to present day non-monsoon months. Such a climate scenario is indeed catastrophic and if persisted for several thousand years could easily convert large monsoon-fed perennial rivers to ephemeral or even dry ones.

## Climate-culture relationship at Harappan Bhirrana

The climate reconstruction at Bhirrana demonstrates that some of the Harappan settlements in the Ghaggar-Hakra valley are the oldest in India and probably developed at least by the ninth millennium BP over a vast tract of arid/semi-arid regions of NW India and Pakistan. The Ghaggar (in India)-Hakra (in Pakistan) river, referred to as mythical Vedic river ‘Saraswati’ (Fig. 1A) originates in the Siwalik hills, ephemeral in the upper part with dry river bed running downstream through the Thar desert to Rann of Kachchh in Gujarat^3^. More than 500 sites of Harappan settlements have been discovered in this belt during the last hundred years. Of these several sites both in India viz. Kalibangan, Kunal, Bhirrana, Farmana, Girawad^7^,^9^,^31^,^33^,^69^ and Pakistan viz. Jalilpur, Mehrgarh in Baluchistan, Rehman Dheri in Gomal plains^29^,^69^,^70^ have revealed early Hakra levels of occupation preceding the main Harappan period. We infer that monsoon intensification from 9 ka onwards transformed the now dried up Ghaggar-Hakra into mighty rivers along which the early Harappan settlements flourished. That the river Ghaggar had sufficient water during the Hakra period is also attested by the faunal analysis. Frequency of occurrence of aquatic fauna like freshwater fish bones, turtle shells and domestic buffalo in these early levels of trench YF-2 is higher (compared to early or mature Harappan periods; SI) indicating a relatively wetter environment.

Study of fluvial morphodynamics coupled with detrital zircon analysis of river channel sands indicated presence of a more energetic fluvial regime before 5 ka across the entire Harappan landscape, stabilized alluvial systems during early Harappan (5.2–4.6 ka BP) and drying up of many river channels during post-Harappan period^3^. Consequently floodplain agriculture helped in the expansion of the Harappan civilization which diminished as the monsoon waned during the late Holocene. Interestingly, the large scale droughts at ~8.2 and ~4.1 ka BP, recorded in the two lake records of Riwasa and Kotla Dahar of Haryana^5^,^6^ correspond to the base of early Harappan and middle part of mature Harappan period at Bhirrana. These events were not local, extended from the Mediterranean through Mesopotamia to China and also are recorded as dust spike in Tibetan ice cores^71^,^72^,^73^. Yet the settlements survived and evolved at several sites of Ghaggar-Hakra belt including at Bhirrana. The climate data and chronology of Bhirrana suggest that not only the Harappan civilization originated during the 8–9^th^ millennium BP, it continued and flourished in the face of overall declining rainfall throughout the middle to late Holocene period^11^,^74^. It is difficult to point to one single cause that drove the Harappan decline although diverse suggestions from Aryan invasion, to catastrophic flood or droughts, change in monsoon and river dynamics, sea-levels, trade decline^2^,^3^,^73^,^74^,^75^,^76^,^77^,^78^,^79^ to increased societal violence and spread of infectious diseases^26^ have been proposed. The continued survival of Harappans at Bhirrana suggests adaptation to at least one detrimental factor that is monsoon change. Although direct paleobotanical data from Bhirrana does not exist, archeobotanical study from nearby Farmana excavation, located ~100 km SW of Bhirrana clearly indicated change in crop pattern through cultural levels. At Farmana, compared to early levels a dramatic decrease in both ubiquity (from 61% to 20%) and seed density (1.5% to 0.7%) in wheat and barley in the later Harappan period has been documented. The study also indicates increasing dependence on summer crops like millet and has been inferred as a direct consequence of lesser rainfall^80^. Such pattern have also been found elsewhere in Indus valley where the Harappans shifted their crop patterns from the large-grained cereals like wheat and barley during the early part of intensified monsoon to drought-resistant species of small millets and rice in the later part of declining monsoon and thereby changed their subsistence strategy^16^,^81^. Because these later crops generally have much lower yield, the organized large storage system of mature Harappan period was abandoned giving rise to smaller more individual household based crop processing and storage system and could act as catalyst for the de-urbanisation of the Harappan civilization rather than an abrupt collapse as suggested by many workers^82^,^83^,^84^,^85^. Our study suggests possibility of a direct connect between climate, agriculture and subsistence pattern during the Harappan civilization.

## Additional Information

**How to cite this article**: Sarkar, A. *et al.* Oxygen isotope in archaeological bioapatites from India: Implications to climate change and decline of Bronze Age Harappan civilization. *Sci. Rep.* **6**, 26555; doi: 10.1038/srep26555 (2016).

## Supplementary Material

Supplementary Information

## References

[b1] Mughal M. R. The decline of the Indus civilization and the Late Harappan period in the Indus valley. Lahore Museum Bulletin 3, 1–17 (1990).

[b2] Possehl G. L. The Indus Civilization: A Contemporary Perspective (Altamira Press Lanham: MD,, 2002).

[b3] Giosan L. *et al.* Fluvial landscapes of the Harappan civilization. Procds. Nat. Acad. Sc. 109, E1688–E1694 (2012). 10.1073/pnas.1112743109 PMC3387054 22645375

[b4] Kenoyer M. Changing Perspectives of the Indus Civilisation: New Discoveries and Challenges. Puratattva 41, 1–18 (2011).

[b5] Dixit Y., Hodell D. A., Petrie C. A. & Sinha R. Abrupt weakening of the Indian summer monsoon at 8.2 kyr B P. Earth Planet. Sc. Lett. 391, 16–23 (2014).

[b6] Dixit Y., Hodell D. A. & Petrie C. A. Abrupt weakening of the summer monsoon in northwest India ~4100 yr ago. Geology 42, 339–342 (2014).

[b7] Shinde V. S., Osada T., Useugi A. & Kumar M. *A report on the excavations at Farmana 2007*–*08*, Occasional papers 6. Linguistics Archaeology and the Human past, Japan Research Institute for Humanity and Research (2008).

[b8] Dikshit K. N. Origin of Early Harappan cultures in the Sarasvati Valley: Recent Archaeological Evidence and Radiometric dates. Jour. Ind. Ocean Arch. 9, 87–141 (2013).

[b9] Nath A. *Excavations at Rakhigarhi 1997–98 to 1999–2000*, Archaeological Survey of India report (2015).

[b10] Singh G., Joshi R. D., Chopra S. K. & Singh A. B. Late Quaternary history of vegetation and climate in the Rajasthan Desert India. Phil. Tran. Royal Soc. Lond. 267, 467–501 (1974).

[b11] Enzel Y. *et al.* High-resolution Holocene environmental changes in the Thar Desert northwestern India. Science 284, 125–128 (1999). 10102808 10.1126/science.284.5411.125

[b12] Staubwasser M., Sirocko F., Grootes P. M. & Erlenkeuser H. South Asian monsoon climate change and radiocarbon in the Arabian Sea during early and middle Holocene Paleoceanography 17(4), 1063–1074 (2002).

[b13] Wright R. P., Bryson R. & Schuldenrein J. Water supply and history: Harappa and the Beas regional survey. Antiquity 8, 37–48 (2008).

[b14] Schuldenrein J. Geoarchaeological perspectives on the Harappan sites of south Asia In: Indian Archaeology in Retrospect (Protohistory): Archaeology of the Harappan Civilization (ed. Settar S. & Korisettar R.) Ch. 2, 47–80 (Indian Council of Historical Research, 2002).

[b15] MacDonald G. Potential influence of the Pacific Ocean on the Indian summer monsoon and Harappan decline Quat. Int. 299, 140–148 (2011).

[b16] Madella M. & Fuller D. Q. Palaeoecology and the Harappan Civilisation of South Asia: a reconsideration. Quat. Sci. Rev. 25, 1283–1301 (2006).

[b17] Rao L. S., Sahu N. B., Sahu P., Shastry U. A. & Diwan S. New light on the excavation of Harappan settlement at Bhirrana. Puratattva 35, 67–75 (2005).

[b18] Mani B. R. Kashmir Neolithic and Early Harappan: A Linkage. Pragdhara 18, 229–247 (2008).

[b19] Kenoyer J. M. Ancient Cities of the Indus Valley Civilization (Oxford University Press: Oxford,, 1998).

[b20] Dikshit K. N. & Mani B. R. Origin of Early Harappan Cultures: A Review. In *International Conference on Harappan Archaeology*, Chandigarh October, 27–29 (2012).

[b21] Shaffer J. G. The Indus Valley, Baluchistan and Helmand Traditions: Neolithic Through Bronze Age In Chronologies in Old World Archaeology (ed. Ehrich R. W.), I 441–464, II:425–446 (University of Chicago Press, 1992).

[b22] Kenoyer M. & Meadow R. H. Inscribed Objects from Harappa Excavations 1986–2007. In Corpus of Indus Seals and Inscriptions 3: New material untraced objects and collections outside India and Pakistan Part 1: Mohenjo-daro and Harappa (eds. Parpola A., Pande B. M., Koskikallio P.). *Mem. Arch. Surv. Ind.* 96, xliv–lviii (2010).

[b23] Wright R. P. The Ancient Indus: Urbanism Economy and Society (Cambridge University Press, 2010).

[b24] Meadow R. H. Animal domestication in the Middle East: A revised view from the Eastern Margin In: Harappan Civilization: A Contemporary Perspective (ed. Possehl G. L., Oxford & I. B. H., New Delhi, 1991).

[b25] Kenoyer J. M. Trade and technology of the Indus Valley: new insights from Harappa Pakistan. World Archaeology 29, 262–280 (1997).

[b26] Schug G. R. *et al.* Infection Disease and Biosocial Processes at the End of the Indus Civilization PLoS ONE 8(12), e84814 (2013). 24358372 10.1371/journal.pone.0084814 PMC3866234

[b27] Misra V. N. Climate a factor in the rise and fall of the Indus Civilization: Evidence from Rajasthan and Beyond In: Frontiers of the Indus Civilization (ed. Lal B. B. & Gupta S. P.), 461–490 (1984).

[b28] Mughal M. R. Ancient Cholistan: Archaeology and Architecture, (Feroz sons, Lahore, 1997).

[b29] Mughal M. R. New evidence for the Early Harappan Culture from Jalilpur, Pakistan. Archaeology 27(2), 106–113 (1974).

[b30] Bhan K. Late Harappan Gujarat. Eastern Anthropol. 45, 173–192 (1992).

[b31] Kumar M. Linguistics Archaeology and the Human Past (eds. Osada T. & Uesugi A.), Research Institute for Humanity and Nature, 1–75 (Nakanishi Printing Co. Ltd : Kyoto,, 2009).

[b32] Lal B. B., Thapar B. K., Joshi P. & Bala M. Excavations at Kalibangan: The Early Harappans (1960–1969), Mem. Arch. Surv. Ind. 98 (2003).

[b33] Nath A. Rakhigarhi 1999–2000. Puratattva 31, 43–46 (2001).

[b34] Rao L. S. Settlement Pattern of the Predecessors of the Early Harappans at Bhirrana District, Fatehabad Haryana. Man and Environment 31, 33–42 (2006).

[b35] Shinde V. *et al.* Exploration in the Ghaggar basin and Excavations at Girawad Farmana (Rohtak District) and Mitathal (Bhiwani District) Haryana India In Linguistiucs Archaeology and the Human Past, Occasional Paper 3 Indus Project (eds. Osada T. & Uesugi A.) 77–158, (Research Institute for Humanity and Nature, 2008).

[b36] Shinde V., Osada T., Uesugi A. & Kumar M. Harappan Necropolis at Farmana in the Ghaggar Basin. (Indian Archaeological Society, New Delhi, 2010).

[b37] Deshpande-Mukherjee A. A preliminary report on the ongoing archaeozoological studies at the Harappan site of Bhirrana dist Fathebad Haryana. Puratattva 42, 202–211 (2012).

[b38] Schmid E. Atlas of animal bones for prehistorians archaeologists and Quaternary geologists, (Elsevier: Amsterdam,, 1972).

[b39] Deshpande-Mukherjee A., Sen A. & Rao L. S. Human Animal Interactions during the Harappan Period in the Ghaggar Region of Northern India Insights from Bhirrana In Bones and Identity: Reconstructing Social and Cultural landscapes in the Archaeozoology of south west Asia, Proc. 11^th^ ASWA Haifa Israel (eds. Marom N., Yeshurun R., Weissbrod L. & Bar-Oz G., Oxbow, 2016 in press).

[b40] Sen A. Role of the fauna in the cultural economy during the Mature Harappan Period at Bhirrana Dist Fatehbad Haryana, Unpub. Masters thesis, (Deccan College Post Graduate and Research Institute, Pune, 2012).

[b41] Elliott J. C. Calcium phosphate biominerals In Phosphates: geochemical geobiological and material importance (eds. Kohn M. J., Rakovan J. & Hughes J. M.) Rev. Mineral. Geochem. 48, 427–453 (2002).

[b42] Luz B. & Kolodny Y. Oxygen isotope variations in phosphate of biogenic apatites: Mammal teeth and bones. Earth Planet. Sc. Lett. 75, 29–36 (1985).

[b43] D’Angela D. & Longinelli A. Oxygen isotopic composition of fossil mammal bones of Holocene age: Palaeoclimatological considerations. Chem. Geol. 103, 171–179 (1993).

[b44] Tütken T., Pfretzschner H. U., Vennemann T. W., Sun G. & Wang Y. D. Paleobiology and skeletochronology of Jurassic dinosaurs: implications from the histology and oxygen isotope compositions of bones *Palaeogeogr*. Palaeoclimat. Palaeoecol. 206, 217–238 (2004).

[b45] Bentaleb I. *et al.* Rhinocerotid tooth enamel ^18^O/^16^O variability between 23 and 12 Ma in southwestern France. Comptes. Rendus. Geoscience 338, 172–179 (2006).

[b46] Pflug K. P., Schuster K. D., Pichotka J. P. & Forstel H. Fractionation effects of oxygen isotopes in mammals In: *Stable Isotopes: Proceedings of the Third International Conference* (ed. Klein, E.R. and Klein, P.D.) 553–561 (1979).

[b47] Bryant J. D. & Froelich P. N. A model of oxygen isotope fractionation in body water of large mammals. Geochim. Cosmochim. Acta 59, 4523–4537 (1995).

[b48] Amiot R. *et al.* Oxygen isotopes from biogenic apatites suggest widespread endothermy in Cretaceous dinosaurs. Earth Planet. Sc. Lett. 246, 41–54 (2004).

[b49] Longinelli A. Oxygen isotopes in mammal bone phosphate: a new tool for paleohydrological and paleoclimatological research? Geochim. Cosmochim. Acta 48, 385–390 (1984).

[b50] Luz B., Kolodny Y. & Horowitz M. Fractionation of oxygen isotopes between mammalian bone-phosphate and environmental drinking water. Geochim. Cosmochim. Acta 48, 1689–1693 (1984).

[b51] Bryant J. D., Koch P. L., Froelich P. N., Showers W. J. & Genna B. J. Oxygen isotope partitioning between phosphate and carbonate in mammalian apatite Geochim. Cosmochim. Acta 60, 5145–5148 (1996).

[b52] Levin N. E. *et al.* A stable isotope aridity index for terrestrial environments. Procds. Nat. Acad. Sc. 103, 11201–11205 (2006). 10.1073/pnas.0604719103 PMC1544065 16840554

[b53] Fricke H. C. & O’Neil J. R. Inter- and intra-tooth variation in the oxygen isotope composition of mammalian tooth enamel phosphate: implications for palaeoclimatological and palaeobiological research. Palaeogeog. Palaeoclim. Palaeoecol. 126, 91–99 (1996).

[b54] Dettman D. L. *et al.* Seasonal stable isotope evidence for a strong Asian monsoon throughout the past 10.7 my. Geology 29, 31–34 (2001).

[b55] Balasse M., Smith A. B., Ambrose S. H. & Leigh S. R. Determining sheep birth seasonality by analysis of tooth enamel oxygen isotope ratios: the Late Stone Age site of Kasteelberg (South Africa). Jour. Archaeol. Sc. 30, 205–215 (2003).

[b56] Sarkar A., Ramesh R., Bhattacharya S. K. & Rajagopalan G. Oxygen isotope evidence for a stronger winter monsoon current during the last glaciation. Nature 343, 549–551 (1990).

[b57] Gupta A. K., Anderson D. M. & Overpeck J. T. Abrupt changes in the Asian southwest monsoon during the Holocene and their links to the North Atlantic Ocean. Nature 421, 354–357 (2003). 12540924 10.1038/nature01340

[b58] Kutzbach J. E. & Street-Perott F. A. Milankovitch forcing of fluctuations in the level of tropical lakes from 18 to 0 kyr BP. Nature 317, 130–134 (1985).

[b59] Overpeck J., Anderson D., Trumbore S. & Prell W. The southwest Indian Monsoon over the last 18 000 years. Clim. Dyn. 12, 213–225 (1996).

[b60] Duplessy J. C. Glacial to interglacial contrast in the northern Indian Ocean. Nature 295, 494–498 (1982).

[b61] Fleitmann D. *et al.* Holocene ITCZ and Indian monsoon dynamics recorded in stalagmites from Oman and Yemen (Socotra). Quat. Sci. Rev. 26, 170–188 (2007).

[b62] Bookhagen B. & Burbank D. W. Topography relief and TRMM-derived rainfall variations along the Himalaya. Geophys. Res. Lett. 33, L08405 (2006).

[b63] Sarkar A. *et al.* Evolution of Ganges–Brahmaputra western delta plain: Clues from sedimentology and carbon isotope. Quat. Sc. Rev. 28, 2564–2581 (2009).

[b64] Anand P. *et al.* Coupled sea surface temperature–seawater δ^18^O reconstructions in the Arabian Sea at the millennial scale for the last 35 ka. Paleoceanography 23, PA4207 (2008).

[b65] Zhang J. W. *et al.* Holocene monsoon climate documented by oxygen and carbon isotopes from lake sediments and peat bogs in China: a review and synthesis. Quat. Sci. Rev. 30, 1973–1987 (2011).

[b66] Cai Y. *et al.* The Holocene Indian monsoon variability over the southern Tibetan Plateau and its teleconnections Earth Planet. Sc. Lett. 335–336, 135–144 (2012).

[b67] Sengupta S. & Sarkar A. Stable isotope evidence of dual (Arabian Sea and Bay of Bengal) vapor sources in monsoonal precipitation over north India. Earth Planet. Sc. Lett. 250, 511–521 (2006).

[b68] Yurtsever Y. & Gat J. Atmospheric waters In: Stable Isotope Hydrology: Deuterium and Oxygen-18 in the Water Cycle Tech Rep Ser. 210 (eds. Gat J. R. & Gonfiantini R.), 103–142, International Atomic Energy Agency: Vienna, (1981).

[b69] Khatri J. S. & Acharya M. Kunal: A new Indus Saraswati site. Puratattva 25, 84–86 (1994–95).

[b70] Durrani F. A. *Excavations in the Gomal valley: Rehman Dheri Excavation Report,* Issue 1 Peshawar: University of Peshawar (1988).

[b71] Jarrige J. F. *et al.* (ed.) *Mehrgarh field reports 1974–1995: From Neolithic Times to the Indus Civilisation*, Karachi, Department of culture and tourism, Government of Sindh (1995).

[b72] Weiss H. Quantifying collapse: The late third millennium Khabur Plains In: Seven generations since the fall of Akkad, (ed. Weiss H.), 1–24, (Wiesbaden Harrassowitz, Verlag, 2012).

[b73] Thompson L. G. *et al.* Kilimanjaro ice core records: Evidence of Holocene cli- mate change in tropical Africa. Science 298, 589–593 (2002). 12386332 10.1126/science.1073198

[b74] Weiss H. & Bradley R. S. Archaeology: What drives societal collapse? Science 291, 609–610 (2001). 11158667 10.1126/science.1058775

[b75] Shaffer J. G. & Liechtenstein D. A. Ethnicity and change in the Indus Valley Cultural Tradition In: Old Problems and New Perspectives in South Asian Archaeology (ed. Kenoyer J. M.), 117–126 (Wisconsin Archaeological Reports, Madison, 1989).

[b76] Lahiri N. Decline and Fall of the Indus Civilization (Permanent Black, Delhi, 2000).

[b77] Ratnagar S. Understanding Harappa: Civilization in the Greater Indus Valley (Tulika Books, New Delhi, 2006).

[b78] McIntosh J. R. The Ancient Indus Valley: New Perspectives (ABC-CLIO, Santa Barbara, 2007).

[b79] Clift P. D. *et al.* U-Pb zircon dating evidence for a Pleistocene Sarasvati River and capture of the Yamuna River. Geology 40, 211–214 (2012).

[b80] Weber S. A., Kashyap A. & Mounce L. Archaeobotany at Farmana: New Insights into Harappan Plant Use Strategies. In: Excavations at Farmana. (eds. Shinde V. S., Osada T. & Kumar M.), 808–823 (RIHN, Nakanish Printing, Kyoto, 2011).

[b81] Pokharia A. K., Kharakwal J. S. & Srivastava A. Archaeobotanical evidence of millets in the Indian subcontinent with some observations on their role in the Indus civilization. Jour. Archaeol. Sc. 42, 442–455 (2014).

[b82] Fuller D. Q. & Madella M. Issues in Harappan archaeobotany: retrospect and prospect. In: Indian Archaeology in Retrospect. *Protohistory*, vol. II. (eds. Settar S. & Korisettar R.), 317–390 (Manohar Publishers, New Delhi, 2001).

[b83] Weber S. A. Archaeobotany at Harappa: indications for change In Indus ethnobiology: new perspectives from the field, (eds. Weber S. A. & Belcher W. R.), 175–198 (Lexington Books Lanham, 2003).

[b84] Fuller D. Q. & Stevens C. J. Agriculture and the development of complex societies In From Foragers to Farmers: Papers in honour of Gordon C Hillman (eds. Fairbairn A. & Weiss E.) 37–57 (Oxford: Oxbow Books, 2009).

[b85] Weber S., Kashyap A. & Harriman D. Does size matter: the role and significance of cereal grains in the Indus civilization. Archaeol. Anthrop. Sc. 2, 35–43 (2010).

