# Supplementary material for: Oxygen isotope in archaeological bioapatites from India: Implications to climate change and decline of Bronze Age Harappan civilization
Source: Sci Rep. 2016 May 25;6:26555. doi: 10.1038/srep26555 (PMC4879637; doi:10.1038/srep26555)
Supplement: Supplementary Information [file srep26555-s1.doc]

**Supplementary Information**

‘Oxygen isotope in archaeological bioapatites from India: Implications to climate change and decline of Bronze Age Harappan civilization’ byAnindya Sarkar, Arati Deshpande Mukherjee, M. K. Bera, B. Das, Navin Juyal, P. Morthekai, R.D. Deshpande, V. Shinde and L. S. Rao

**A. Archeological information of Bhirrana site**

**1) Chronology of Indus valley civilization**

Two major archaeological chronologies of Harappan (Indus) civilization in South Asia (compiled based on data from previously published papers of Kenoyer, 1998; Possehl 2002; Madella and Fuller, 2006)

|  | Kenoyer (2011)# | Possehl (2002)@ |  |
| --- | --- | --- | --- |
| Phase/Period | Cal Years BP | Cal Years BP | Stage |
| Early Harappan/Ravi Phase: 1A/B | 5700–2800 | 9000–6300 | Early village farming communities and pastoral societies |
| Early Harappan/Kot Diji Phase:2 | 4800–4600 | 6300–5200 | Advanced village farming communities and pastoral societies |
| Harappan Phase:3A | 4600–4450 | 5200-4600 | Early Harappan |
| Harappan Phase:3B | 4450-4200 | 4600–4500 | Early Harappan/Mature Harappan Transition |
| Harappan Phase:3C | 4200–3900 | 4500–3900 | Mature Harappan |
| Harappan/Late Harappan Transitional | 3900–3700 | 3900–3000 | Post-urban Harappan |
| Late Harappan (Cemetery H) | 3700–3300 | 3000–2500 | Early Iron Age of India and Painted Grey Ware |

# Proposed based on the stratigraphy of Harappa (Punjab), Pakistan

@ Proposed based on larger regional compilation in south Asia

**2) Radiocarbon and OSL chronology in Bhirrana trenches**

Uncalibrated and calibrated radiocarbon dates of the charcoal samples analysed from the Bhirrana after the excavation of 2005 (compiled based on data from previously published papers of Mani, 2008#); OSL dates are from present work@ (see below).

| Site/  Sample no. | Trench | Lab. No. | Depth  (m) | 14C age±1  year BC | Calibrated age±1  year BC | | Calibrated age±1  year BP | | OSL  Date  Cal year BP | |
| --- | --- | --- | --- | --- | --- | --- | --- | --- | --- | --- |
|  |  |  |  |  | Max. | Min. | Max. | Min. | Max. | Min. |
| BRN-1# | A-1 | BS-2308 | 0.45-.50 | 1350 ± 200 | 1876 | 1324 | 3826 | 3274 |  |  |
| BRN-Pot-1@ | YF-2 | PRL-43 | 0.42-0.46 |  |  |  |  |  | 5120 | 4520 |
| BRN-3# | ZE-10 | BS-2310 | 1.25 | 1240 ± 160 | 1679 | 1264 | 3629 | 3214 |  |  |
| BRN-Pot-2@ | YF-2 | PRL-143 | 1.43-1.45 |  |  |  |  |  | 6185 | 5695 |
| BRN-5# | ZE-10 | BS-2318 | 1.42 | 4170 ± 250 | 5336 | 4721 | 7286 | 6671 |  |  |
| BRN-6# | A-1 | BS-2333 | 2.95 | 5640 ± 240 | 6647 | 6221 | 8597 | 8171 |  |  |

14C ages were determined by conventional method [14C/12C ratios normalized assuming organic matter 13C = –25.0 ‰ (Stuiver and Polach 1977)] and were then calibrated to get calendar ages. Calibration was carried out by the probability method of OxCal v 4.1 (Bronk Ramsey, 2009) and the IntCal09 data set (Reimer et al., 2009). All 14C ages are based on a half life of 5.730± 40 year (for detail methods see Sukumar et al., Nature, 1993; Goyal et al., 2013). OSL methods described below.

**3) Schematic E-W cross section of the trench YF-2 depicting the cultural levels at Bhirrana** (Rao et al., 2005; Dikshit, 2013; with permission from Archaeological Survey of India)

**
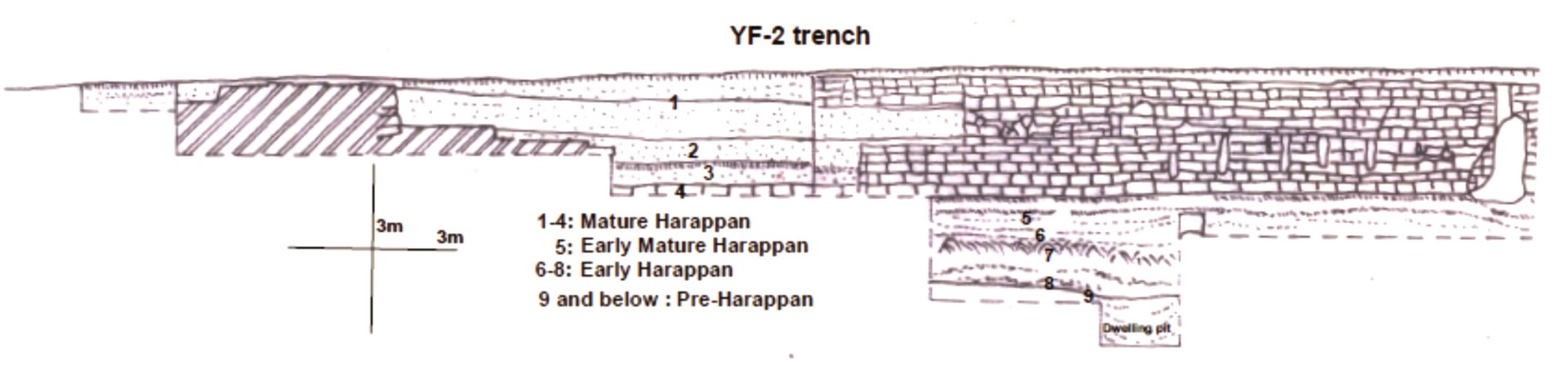
**

**4) Cultural stratigraphy of Bhirrana settlement (compiled based on data from previously published papers of Rao et al., 2004-05; Dikshit and Mani, 2012)**

| **Period** | **Cultural levels** | **Year BP**  (based on radiocarbon ages in different trenches) | **Attributes** |
| --- | --- | --- | --- |
| II B | Mature Harappan culture | 3000-1800 BCE | Fully developed house complexes contain painted ceramics which included geometric, floral and faunal motifs. Incised figure of a dancing girl closely resembling the famous bronze dancing girl from Mohenjo-daro. Antiquities typical of the Mature Harappan period were recovered such as steatite seals, beads of semi-precious stone, shell and terracotta, animal figurines, bangles of faience, shell, copper bangles, chisels, rings, rods, stylised terracotta horns with symbolic head painted in black. |
| II A | Early Mature Harappan culture | 4500-3000 BCE | Beginning of fortification wall, house-complexes, streets and lanes |
| IB | Early Harappan culture | 6000-4500 BCE | Settlement expanded and the entire site came under occupation. The houses were built of mud bricks in the ratio of 3:2:1 and measured 45x30x15cm; 42x28x 14cm and 39x36x 13cm. Yielded terracotta figurines, arrow heads, rings and bangles of copper, beads of carnelian, jasper, shell, bull figurines, chert blades, terracotta bangle. |
| I A | Hakra ware culture | 7500-6000 BCE | Earliest cultural phase at Bhirrana, primarily identified from the ceramics quite similar to those identified from sites in Cholistan. The ceramics comprise mud appliqué ware, incised ware, Bi-chrome ware, tan slipped ware, blackburnished ware, brown on buff ware, simple red ware of medium fabric with common shapes like vase, bowl and cup. Also characterized by its subterranean dwelling pits Antiquities from the dwelling pits included beads of semiprecious stones like carnelian, agate, terracotta bangles, unbaked triangular clay cakes, querns, crucible, chert blade, crucible fragments with molten copper. |

**5) Archeological artefacts from different cultural levels at Bhirrana (Rao et al., 2004-05; Mani, 2008; permission taken from Archeological Survey of India)**

**
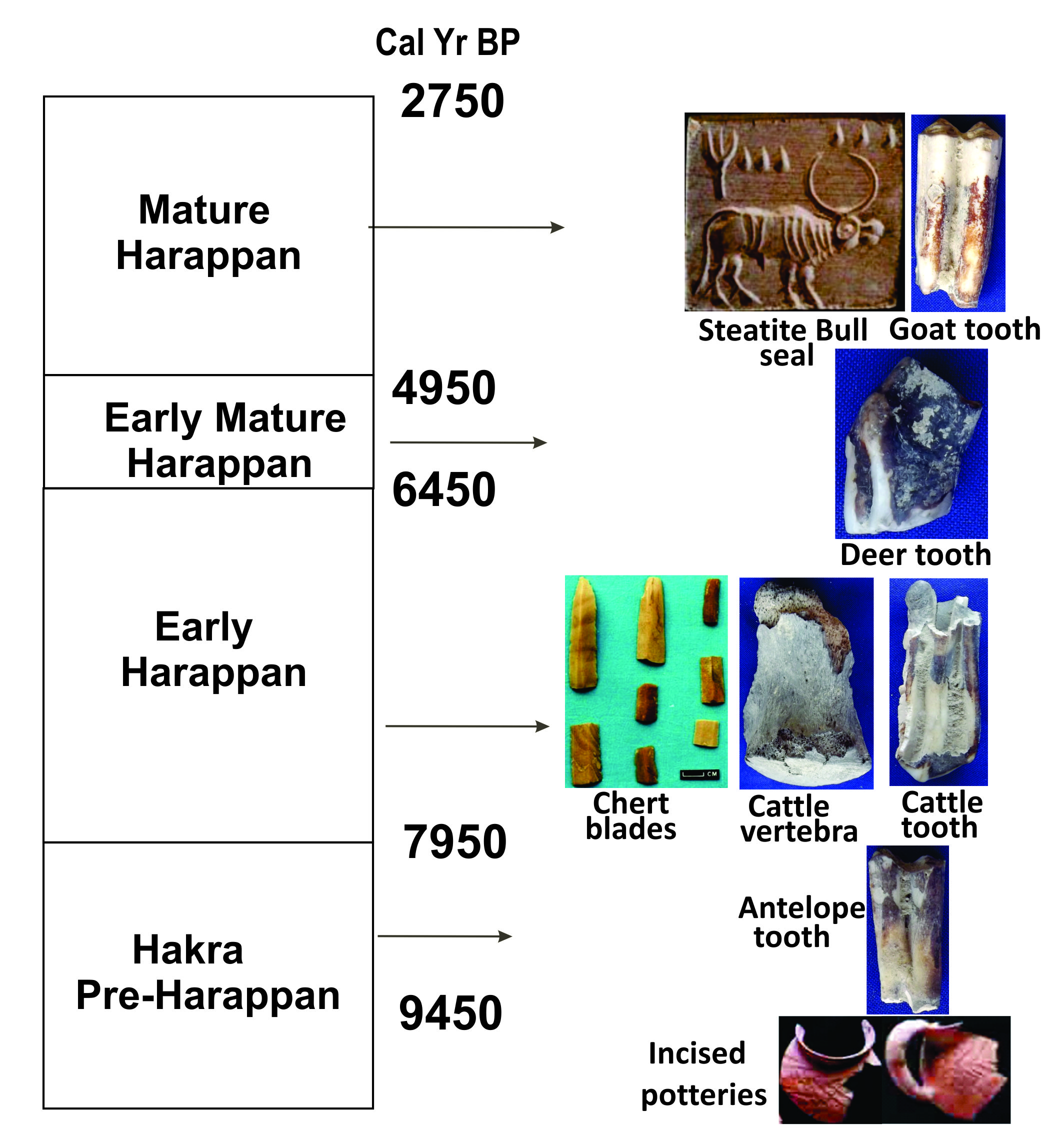
**

**B. Experimental methods**

**Supplementary Method 1:**

*Optically stimulated luminescence (OSL) dating of potteries*

The pottery samples were collected from the corresponding sections (Fig. 1). The outer 1-2 mm layers were physically scraped under the subdued red light. The unexposed inner part of the pottery was gently crushed using acetone in an agate mortar. The powdered samples were treated with 1 N HCl and 40 % H2O2 to remove carbonates and organic materials respectively. Following this the samples were deflocculated by using 0.01 N sodium oxalate and the clay minerals were removed from the solution. The samples were then suspended in an alcohol column and the ~ 4-11 μm grain fractions were separated using Stokes’ times of 1.5 and 15 minutes. The separated fraction was re-suspended in alcohol and ~1 ml volumes were pipetted on to 9.65 mm aluminum discs and dried at ~45°C (Singhvi et al., 2001).

The Infrared Stimulated Luminescence (IRSL) measurements were carried out in automated Risoe TL/OSL Reader DA-20 (Boetter-Jensen et al., 2003). It is equipped with a calibrated β-particles dose rate 0.09 Gy.s-1 to the fine grains of feldspar mounted on Al discs. The samples were stimulated by IR LEDs (870 ± 40 nm) and the luminescence photons were detected in the range of 395 ± 50 nm using PMT (EMI9235QB) and the combination of optical filters of Corning 7-59 (4 mm) and BG-39 (2 mm). A preheat value of 260°C held of 10 s was used. The dose response curve of BRN 5 - 6 (42-46) is shown in Fig. 1 and a typical shine down curve is shown in figure1Sa (inset). Recycling ratios were unity within 5 % error and the recuperation was less than 1 %. Equivalent doses (De’s) were evaluated using single aliquot regenerative dose (SAR) of Murray and Wintle (2003). Around 20 aliquots were measured from each sample which shows insignificant over-dispersion (< 3 %) in the dose distribution (Fig. 2).

In order to estimate the α- efficiency (a-value), six fresh aliquots were bleached in the solar simulator for 6 hours and were irradiated with calibrated 241Am for 30 minutes. The α irradiated aliquots are treated as natural signal and the dose is recovered using the appropriate β-dose using SAR protocol. The a-value (α−efficiency) in IRSL production was calculated by comparing equivalent β-dose and the irradiated α-dose. For radioactivity assay, concentrations of U, Th and K in the sediment matrix has been measured using high pure Ge (HPGe) detector by comparing the photo-peak of the sample against that of corresponding standards (Shukla, 2011). Considering the small size and the thickness of the pottery, it is reasonable to assume that the dose contribution would be predominantly from the sediment and hence only the dose rate from the sediment has been considered for the age estimation. In absence of sediment sample for Early Harappan pottery, we used the average dose rate of the BRN-5 (143-145) and BRN 5-6 (42-46). Details of the dose rate equivalent dose and the ages obtained are given in Table 1.

In order to correct for the fading of feldspar luminescence signal (anomalous fading), the percentage fading rate (g-values; %/decade) were measured (Auclair et al., 2003) and corrected using the method suggested by Huntley-Lamothe (2001).


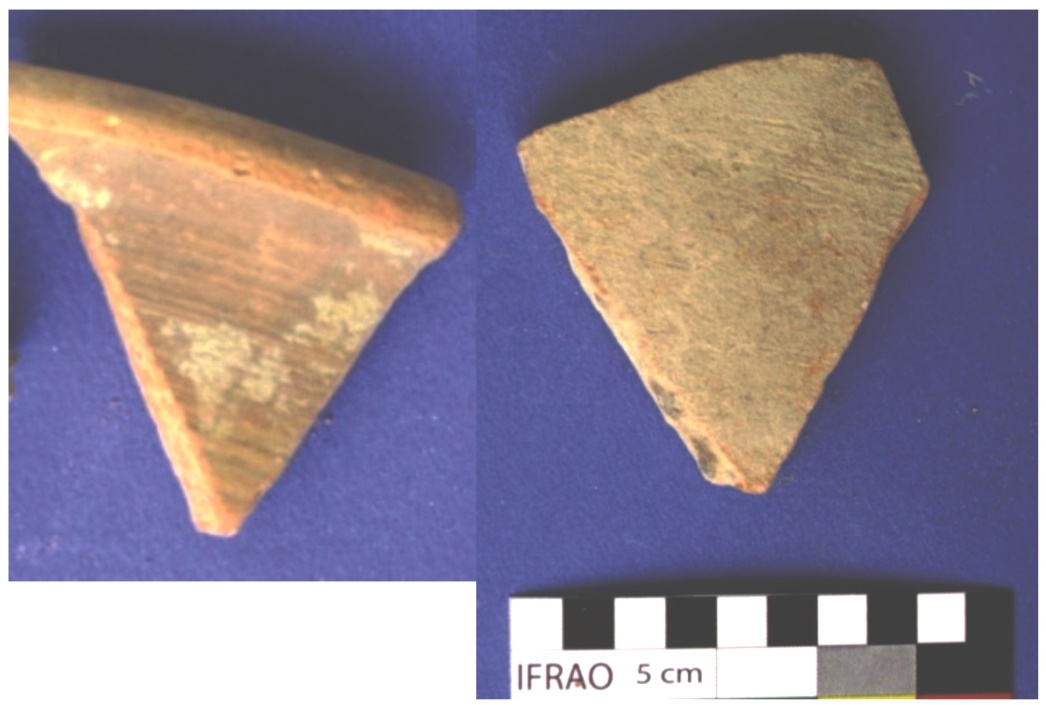


Fig. 1: Pottery fragments (photographs taken by us) from YF-2 trench (left: 42 cm.), right (143 cm)


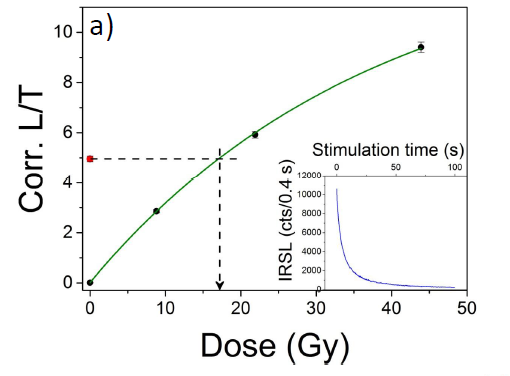


**Fig. 2 Fig. 3**

Fig. 2: The dose response function of BRN 5 (42-46 cm) fitted to single saturating exponential (green line). Sensitivity change corrected IRSL (corr. L/T) has been plotted versus the laboratory administered radiation dose. IRSL shine down curve (natural) of the same sample is shown in the inset.

Fig. 3: Probability and probability density function (PDF) were plotted for the same sample. 15 aliquots were measured for this sample and they were clustered around the mean value of 15.5 Gy.

**Table 1**

Concentrations of U, Th and K, calculated dose rates and the OSL ages of potteries

| **Sample Type/Code/Depth** | **U (ppm)** | **Th (ppm)** | **K (%)** | **a- value** | **Dose rate (Gy/ka)** | **Palaeodose (Gy)** | **g2days-value(%/decade)** | **Corr. Age (years)** |
| --- | --- | --- | --- | --- | --- | --- | --- | --- |
| Pottery, YF-2 trench, BRN 5 (143-145 cm) | 5.1 ± 0.6 | 6.9 ± 1.2 | 1.3 ± 0.2 | 0.036 ± 0.002 | 3.9 ± 0.1 | 17.6 ± 0.8 | 4.3 ± 0.8 | 5940 ± 245 |
| Pottery, YF-2 trench, BRN 5 (42-46 cm) | 7.3 ± 0.9 | 7.1 ± 1.5 | 1.8 ± 0.1 | 0.029 ± 0.003 | 4.9 ± 0.2 | 15.5 ± 0.7 | 6.0 ± 2.0 | 4820 ± 300 |

1. Water content was assumed to 5 ± 2 %
2. Cosmic ray dose rate was assumed to be 150 ± 20 Gy/ka.
3. Grain size used: 4-11 μm

**Supplementary Method 2:**

*Oxygen isotope analysis of bioapatites*

Individual tooth or bone was cleaned by distilled water, surface coatings removed, ultrasonicated, dried and only the surficial enamel part (~0.2-0.4 mm layer) was sampled perpendicular to the entire growth axis by a micro-dental drill to obtain bulk phosphate sample. Extraction of pure phosphate from bioapatite in the form of Ag3PO4 is a necessary pre-requisite for isotopic measurements. The Ag3PO4 was extracted by following the method described in Stephen (2000). Typically 2 mg of bioapatite yields ~1 mg of Ag3PO4. About 300*μ*g of Ag3PO4 was packed into pure silver capsules and loaded onto the automated carousel atop a temperature conversion elemental analyzer (TC-EA). The sample was combusted at ~1450oC and the generated CO was analysed in a Delta PlusXP mass spectrometer via a ConFlo interface. For routine analysis of bioapatites, an inter-laboratory calibration exercise was performed by two standards, namely international NIST 120C Phosphate Rock standard (δ18OSMOW = +22.65 ‰) and Acros Silver Phosphate (ASP) standard (δ18OSMOW = +14.2 ‰) obtained from the KPESIL Isotope Laboratory, University of Kansas. The NIST phosphate rock was chemically treated to precipitate the Ag3PO4 crystals following the method described above. The obtained 18OASP-SMOW value (via NIST) at IIT, Kharagpur is +14.4 ‰ and is in excellent agreement with the value of +14.2 ‰ recommended by KEPSIL. Overall analytical reproducibility was ± 0.2 ‰ similar to obtained elsewhere by both dual inlet and on-line CFIRMS technique. For retrieving paleo-meteoric water value from the Bhirrana teeth and bones we used the general mammal equation, 18OW= 1.0247*δ18Op - 25.02 (Amiot et al. (2004). The relationship is based on global compilation of teeth and bone phosphates of variety of continental mammalian apatites with large range of 18OW­ varying from +5‰ to -25‰, independent of species or genera, exhibiting high degree of correlation between 18Op and 18OW­ and therefore robust.

**Table 2: 18**O ingested water calculated from Tooth and Bone phosphate in Bhirrana trench YF-2

| **Sl. No.** | **Depth (cm)** | **Sample type (Teeth)** | **18Op ‰* (VSMOW)** | **18Ow‰ @**  **(VSMOW)** | **Sl. No.** | **Depth (cm)** | **Sample type (Bone)** | **18Op‰* (VSMOW)** | **18Ow‰@ (VSMOW)** |
| --- | --- | --- | --- | --- | --- | --- | --- | --- | --- |
| 1 | 42 | Goat Tooth | 23.86 | -2.19 | 1 | 42 | Cattle Bone | 22.56 | -2.08 |
| 2 | 42 | Goat Tooth | 22.11 | -3.39 | 2 | 42 | Cattle Bone | 19.57 | -5.16 |
| 3 | 60 | Cattle Molar | 25.91 | 1.37 | 3 | 42 | Cattle Bone | 19.91 | -4.81 |
| 4 | 60 | Cattle Molar | 24.81 | 0.24 | 4 | 133 | Cattle rib | 19.61 | -5.12 |
| 5 | 60 | Goat Molar | 20.76 | -4.32 | 5 | 175 | Cattle Long Bone | 20.83 | -3.87 |
| 6 | 60 | Cattle Molar | 25.91 | 1.37 | 6 | 175 | Cattle Femur shaft | 21.65 | -3.02 |
| 7 | 148 | Deer pre-Molar | 22.73 | -2.53 | 7 | 175 | Cattle Femur shaft | 21.17 | -3.52 |
| 8 | 148 | Deer pre-Molar | 21.27 | -3.81 | 8 | 183 | Cattle Long Bone | 20.60 | -4.10 |
| 9 | 185 | Cattle Tooth fragment | 20.51 | -4.20 | 9 | 195 | Cattle long Bone shaft | 21.10 | -3.59 |
| 10 | 185 | Ruminant tooth fragment | 20.90 | -3.79 | 10 | 230 | Cattle Bone | 19.67 | -5.06 |
| 11 | 185 | Unidentified Tooth fragment | 17.88 | -6.90 | 11 | 240 | Cattle Bone | 19.99 | -4.73 |
| 12 | 195 | Cattle pre-Molar | 20.42 | -4.29 | 12 | 240 | Cattle Bone | 21.73 | -2.94 |
| 13 | 205 | Cattle Molar | 21.05 | -3.64 | 13 | 278 | Cattle rib | 16.41 | -8.42 |
| 14 | 212 | Cattle Molar | 22.54 | -2.10 | 14 | 278 | Cattle rib | 19.24 | -5.51 |
| 15 | 212 | Cattle Molar | 21.78 | -2.89 | 15 | 302 | Cattle long Bone shaft | 19.77 | -4.96 |
| 16 | 212 | Cattle Molar | 21.81 | -2.86 | 16 | 308 | Cattle Long Bone shaft | 15.84 | -9.01 |
| 17 | 300 | Cattle maxillary molar fragment | 21.86 | -2.80 | 17 | 308 | Cattle Long Bone shaft | 18.64 | -6.12 |
| 18 | 300 | Cattle tooth fragment | 18.43 | -6.34 | 18 | 325 | Cattle Vertebral spine | 18.42 | -6.35 |
| 19 | 302 | Cattle Molar | 19.25 | -4.20 | 19 | 330 | Cattle Long Bone splinter | 19.99 | -7.51 |
| 20 | 314 | Antelope Molar | 24.99 | 0.38 |  |  |  |  |  |
| 21 | 314 | Cattle Molar | 24.83 | 0.26 |  |  |  |  |  |
| 22 | 320 | Cattle Molar | 19.94 | -4.78 |  |  |  |  |  |
| 23 | 339 | Goat Molar | 24.04 | -2.07 |  |  |  |  |  |
| 24 | 340 | Cattle pre-Molar | 21.94 | -2.72 |  |  |  |  |  |
| 25 | 340 | Goat mandibular | 22.94 | -2.82 |  |  |  |  |  |
| 26 | 355 | Ruminant tooth fragment | 27.4 | 2.91 |  |  |  |  |  |
| 27 | 355 | Ruminant tooth fragment | 28.22 | 3.75 |  |  |  |  |  |

** 18Op** = ** 18O of tooth enamel /bone phosphate;**

**@ 18Ow = 18O of ingested water (proxy meteoric water), calculated using the taxon specific mammal equations of Bryant and Froelich (1995).**

**Supplementary Method 3:**

*Faunal analysis in trench YF2*

Faunal material recovered from Trench YF2 at Bhirrana through use of systematic recovery techniques such as dry and wet sieving was analysed at the Archaeozoology Laboratory DCPRI, Pune following standard procedures in Archaeozoological analysis. Identification to the species level was carried out by comparison with the reference collection of modern animal skeletons housed within the Archaeozoology lab and by referring to Schimd’s (1972), Hillson (1992) and Prater (1971). For more specific identification between cattle and Nilgai (Joglekar et.al. 1994), sheep and goat (Boessneck, 1969; Prummel and Fisch 1986; Zeder and Lapham 2010), blackbuck (*Antelope cervicapra*), goat (*Capra hircus* ) and sheep (*Ovisaires*) (Pawankar and Thomas 2001) both MNI and NISP estimation were done. The age estimation of individual animal was calculated by studying teeth eruption patterns following Grant (1982) and epiphysal fusion in long bones (Silver, 1963). Bone measurements were taken wherever possible (Driesh 1976). Each bone fragment was carefully scrutinized for traces of human activity such as charring, cut and chop marks, abrasion, polishing, breakage patterns and state of preservation. The faunal remains showed fairly good preservation and in spite of the fragmentation identification to species level was possible for many of the bones. A total of 1039 animal skeletal elements were analysed from all the 4 cultural periods of which total identifiable specimens accounted for n=561.

The analysis revealed the presence of a diverse range of domestic and wild mammals with few birds and fish from trench YF-2 and ZE-10 (Table 3). Faunal evidence strongly suggests the heavy reliance on animal foods by the Bhirrana inhabitants throughout its occupation. Of these the cattle (*Bos/Bubalus*) show maximum exploitation specifically of the domestic cow/ox (*Bos indicus*) in all the four cultural periods followed by that of domestic goat. In the Hakra period i.e. the earliest occupation phase at Bhirrana, Zebu the famed humped variety of *Bos indicus* has been recorded. In this period, the varied wild fauna identified unlike cattle diminishes in the succeeding Early Harappan and Early Mature Harappan periods only to occur once again in the Mature Harappan period. Identification of the domestic buffalo (*Bubalus bubalis*) in the early levels in YF2 further indicates a wet environment. However, there is no definitive trend in the animal abundances that can be related to monsoon vagaries. The only inference that can be made is the river Ghaggar had sufficient water to support aquatic fauna during the Hakra period as attested by the occurrence of freshwater fish bones and turtle shells and is discussed in the text.

**Table 3: Depth wise NISP distribution of identified fauna in trench YF2 at Bhirrana**

| **Depth (cm)** | **B/B** | **BI** | **BB** | **C/O** | **Ch** | **Oa** | **SR** | **Ac** | **Bt** | **Aa** | **Gz** | **Mm** | **Cu** | **P** | **FH** | **BRD** | **Total** |
| --- | --- | --- | --- | --- | --- | --- | --- | --- | --- | --- | --- | --- | --- | --- | --- | --- | --- |
| 42-46 | 9 | 13 |  | 4 | 1 |  |  | 4 |  |  |  |  |  |  |  |  | 31 |
| 58-70 | 28 | 12 |  |  |  | 3 |  |  |  |  |  |  |  |  |  |  | 43 |
| 60-88 | 53 | 17 |  |  |  |  | 3 |  |  |  | 1 |  |  | 1 |  |  | 75 |
| 83-94 | 28 | 34 |  |  |  |  | 5 |  | 2 | 1 |  |  |  |  |  |  | 70 |
| 90-115 | 18 | 31 |  |  |  |  |  | 3 | 2 |  |  |  |  |  |  |  | 54 |
| 125-133 |  | 2 |  |  |  |  |  |  |  |  |  |  |  |  |  |  | 2 |
| 143-162 | 23 | 9 | 1 |  |  | 2 | 2 |  |  |  |  |  |  |  |  |  | 37 |
| 162-175 |  | 1 | 1 | 2 |  |  | 1 |  |  |  |  |  |  |  |  |  | 5 |
| 175-187 | 5 | 2 |  |  |  |  | 1 |  |  |  |  |  |  |  |  |  | 8 |
| 185-195 |  | 1 |  |  |  |  |  |  | 1 |  |  |  |  |  | 1 |  | 3 |
| 195-200 | 1 | 1 |  |  | 5 |  |  |  |  |  |  |  |  |  |  |  | 7 |
| 205-208 | 13 |  |  |  |  |  | 1 |  |  | 1 |  |  |  |  |  |  | 15 |
| 212-230 | 1 | 2 |  |  | 1 |  | 1 |  |  |  |  |  |  |  |  |  | 5 |
| 225-250 | 31 | 8 | 5 |  | 2 |  |  |  | 1 |  |  |  |  |  |  |  | 47 |
| 240-260 | 2 | 5 | 1 | 2 |  |  |  | 1 | 3 |  |  |  |  |  |  |  | 14 |
| 260-278 | 2 | 5 |  |  | 1 |  |  |  |  |  |  |  |  |  |  |  | 8 |
| 278-290 | 4 | 2 |  |  |  | 2 |  |  |  |  | 1 |  |  |  |  |  | 9 |
| 300-326 | 17 | 4 | 1 | 1 | 2 |  | 3 | 2 |  |  |  | 1 | 1 |  | 3 |  | 35 |
| 325-330 | 4 | 2 |  |  |  |  | 4 |  |  |  |  |  |  |  |  |  | 10 |
| 330-335 | 2 |  | 1 |  |  | 1 | 2 |  |  |  |  |  |  |  |  |  | 6 |
| 335-340 | 14 | 4 |  | 2 | 1 |  | 3 | 1 |  | 1 |  |  |  |  |  |  | 25 |
| 340-355 | 30 | 4 |  |  | 1 |  | 4 | 1 |  | 1 |  | 1 |  |  |  | 1 | 43 |
| 351-362 | 2 |  |  | 1 |  |  | 1 |  |  |  |  |  |  |  |  |  | 4 |
| 362-364 |  | 3 |  |  |  |  | 1 |  |  |  |  |  |  |  |  |  | 4 |
| **Total** | 287 | 162 | 10 | 12 | 14 | 8 | 32 | 12 | 9 | 4 | 2 | 2 | 1 | 1 | 4 | 1 | 561 |

**Abbreviations used B/B:** Cattle *(Bos/bubalus);* **BI:** Cow/ox *(Bos indicus),* **BB:** Buffalo  *(Bubalus bubalis***), C/O***:* Goat/sheep *(Capra/Ovis),* **Ch**: Goa*t ( Capra hircus* ), **Oa**: Sheep (*Ovis aries),* **SR**: Small ruminant; **Ac:** Black buck *(Antilope cervicapra),* **Bt**: Nilgai (*Boselaphas tragocamelus),* **Aa***: Spotted deer (Axis axis),* **Cu**: Sambar (*Cervus unicolor* ), **Gz***: (Gazella bennetti),* **Mm***:(Muntiacus muntjack),* **P:** *(Panthera pardus),* **BRD**: Bird*,* **FH:** fish.


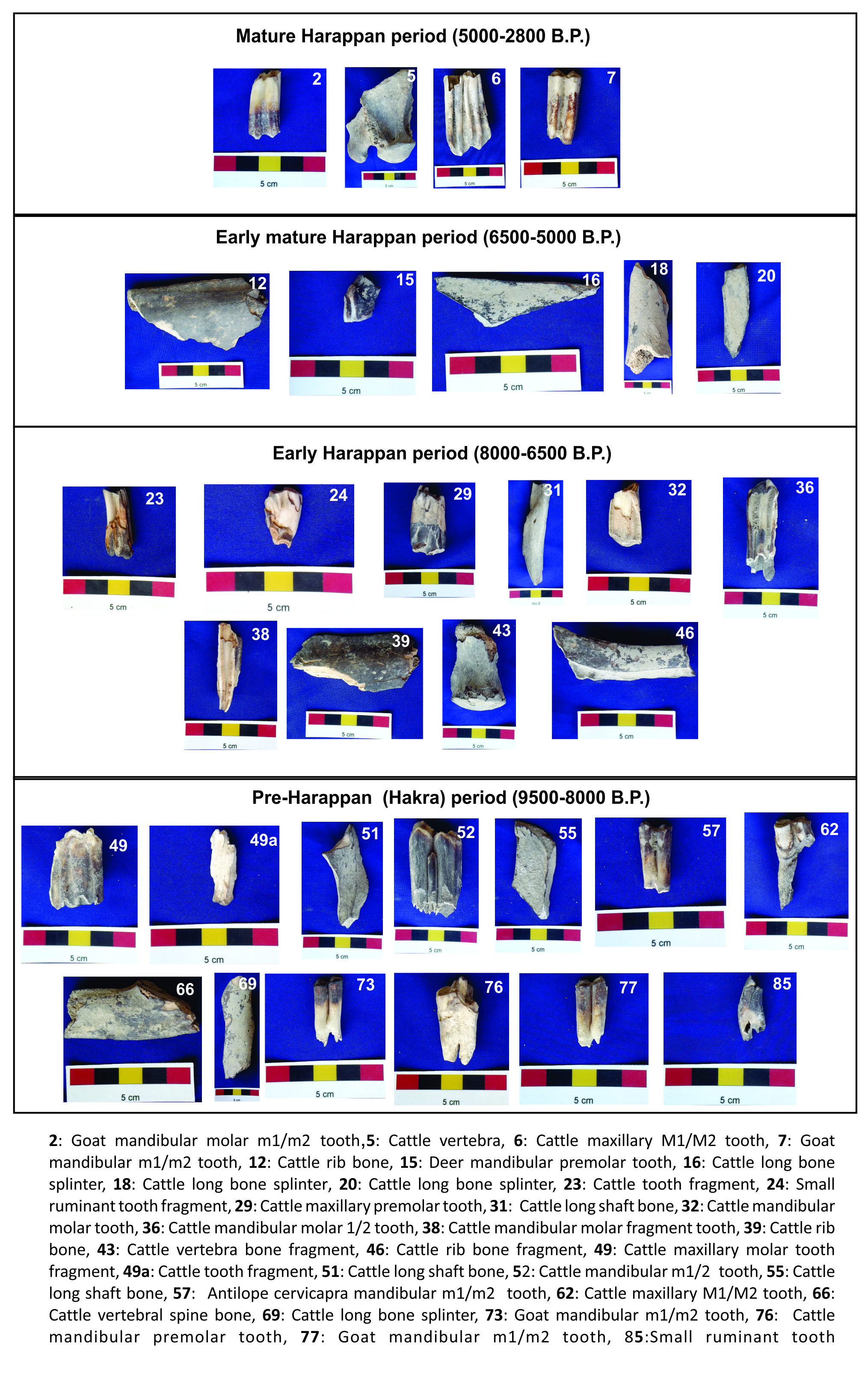


Fig. 4: Representative teeth and bone samples analysed from all

the four cultural levels of Bhirrana (photographs taken by us)

**Supplementary Method 4:**

*Diagenetic investigation of bioapatites*

Carbon-coated polished halves of thin sections up to 300 μm thick were used for the Electron microprobe analysis (EPMA). The sample were analysed for major and minor elements using the Cameca SX 100 microprobe. The spot analysis were done with an accelerating voltage of 15 kV with a beam diameter of 20 micrometer in order to account for the limited stability of bioapatite under the electron beam. Counting times were 20-60s on the peak and 10-40s on the background. Fig. 5 gives the Back Scatter Electron (BSE) images of Ca and P.

**
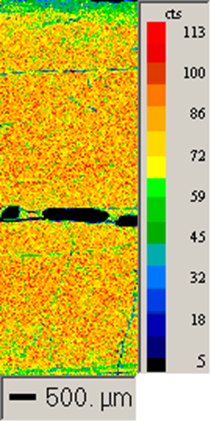

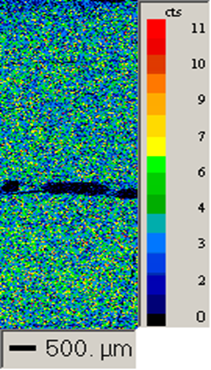
**

Fig. 5: BSE image of bioapatite of mammal bones. Left: Ca; Right: P

(photographs taken by us)

The Ca/P ratio is approximately 1.4 and the CaO/P2O5 ratios are very constant suggesting near-pristine values (Newseley, 1998). The Back Scatter Electron (BSE) images of the samples show uniformity in Ca and P distribution and are indicative of original bioapatite preservation suitable for isotopic analysis.

**References**

Amiot, R., Lécuyer, C., Buffetaut, E., Escarguel, G., Fluteau, F., Martineau, F., 2004. Oxygen isotopes from biogenic apatites suggest widespread endothermy in Cretaceous dinosaurs. Earth and Planetary Science Letters 246, 41–54.

Auclair, M., Lamothe, M. and Huot, S., 2003. Measurement of anomalous fading for feldspar IRSL using SAR. Radiation Measurements 37, 487-492.

Boessneck, J. (1969) Osteological differences between sheep (*Ovis aries* Linne) and goat (*Capra hircus* Linne). In Brothwell. D, and E.S. Higgs (eds) Science and Archaeology, 331-358. London, Thames and Hudson.

Boetter-Jensen, L., Andersen, C. E., Duller, G. A. T., Murray, A. S., 2003. Developments in radiation, stimulation and observation facilities in luminescence measurements. Radiation Measurements 37, 535-541.

Bronk Ramsey C. 2009. Bayesian analysis of radiocarbon dates. Radiocarbon 51(1):337–60.

Driesch, von A, (1976) A guide to measurement of animal bones from archaeological sites, Cambridge , Massachusetts Harvard university Press.

Goyal Pankaj, Pokharia, Anil K., Kharakwal, J.S., Joglekar, P et al., 2013. Subsistence system, paleoecology and 14C chronology at Kanmer, a Harappan site in Gujarat, India. Radiocarbon, 55(1):141-150.

Grant, A. (1982) The use of tooth wear as a guide to the age estimation of domestic ungulates. In B.Willson, C. Grigson and S. Payne (eds.) ageing and sexing animal bones from archaeological sites, 91-108. Oxford :Bar international series 109.

Hillson, S. (1992) Mammal Bones and Teeth ; London : Institute of Archaeology.

Huntley, D. J. and Lamothe, M., 2001. Ubiquity of anomalous fading in K-feldspars and the measurement and correction for it in optical dating. Canadian Journal of Earth Sciences 38, 1093-1106.

Joglekar, P.P., Thomas, P.K., Matsushima, Y. and Pawankar, S.J. (1994) Osteological differences between the forelimbs of Ox (*Bos indicus*), Bufalo (*Bubalus bubalis*) and Nilgai (*Boselaphus tragocamelus*). Journal of Bombay Veternary College 5(1-2) :17-20.

Kenoyer M (2011) Changing Perspectives of the Indus Civilisation: New Discoveries and Challenges Puratattva 41: 1-18.

Madella M, Fuller DQ (2006) Palaeoecology and the Harappan Civilisation of South Asia: a reconsideration Quat Sci Rev 25:1283–1301.

Mani BR. (2008) Kashmir Neolithic and Early Harappan: A Linkage. Pragdhara. 18: 229–247.

Murray, A. S. and Wintle, A. G., 2003. The single aliquot regenerative dose protocol: potential for improvements in reliability. Radiation Measurements 37, 377-381.

Newseley, H. 1989. Fossil bone apatite. Applied Geochemistry 4, 233-245.

Pawankar, S. and Thomas, P.K. (2001) Osteological differenes between Blackbuck (*Antilope cervicapra* ) , Goat (*Capra hircus*) and sheep(*Ovis aries*). Man and Environment 26(1) , 109-126.

Possehl GL (2002) The Indus Civilization: A Contemporary Perspective (Altamira Press Lanham MD), pp.276.

Prummel, W. and Frisch, H. (1986) A guide to the distinction of species, sex and body sides in bones of sheep and goat. Journal of Archaeological Science 13, 567-577.

Prater, S.H. (1971) The book of Indian Animals. Mumbai, Bombay Natural History Society. Oxford University Press.

Rao LS, Sahu NB, Sahu P, Shastry UA, Diwan S (2005) New light on the excavation of Harappan settlement at Bhirrana Puratattva 35:67-75.

Reimer PJ, Baillie MGL, Bard E, Bayliss A, Beck JW et al., 2009. IntCal09 and Marine09 radiocarbon age calibration curves, 0–50,000 years cal BP. Radiocarbon 51(4):1111–50.

Schimd, E. (1972) Atlas of Animal Bones. Amsterdam, Elsevier Publishing company.

Shukla, A.D. 2011. Geochemical and Isotopic studies of some sedimentary sequences of the Vindhayan Super Group, India (Unpublished Ph.D thesis). M.S. University, Baroda.

Silver, I. A. (1963) The Ageing of domestic animals. In D. Brothwell and Higgs, E.S. (eds.) Science and Archaeology 283-302, London: Thames and Hudson.

Singhvi AK, Bluszcz A, Bateman MD, Rao Someshwar. 2001. Luminescence dating of loess-palaeosol sequences cover sands: methodological aspects and palaeoclimatic implications. Earth Science Reviews 54**:** 193 – 211.

Stephan, E. 2000 Oxygen Isotope Analysis of Animal Bone Phosphate: Method Refinement, Influence of Consolidants, and Reconstruction of Palaeotemperatures for Holocene Sites. Journal of Archaeological Science 27: 523–535.

Stuiver M, Polach HA. 1977. Discussion: reporting of 14C data. Radiocarbon 19(3):355–63.

Sukumar, R., Ramesh, R., Pant, R. K., and Rajagopalan, G.: 1993, 'A 613C Record of Late Quaternary Climate from Tropical Peats in Southern India', Nature 364, 703-706.

Zeder, M.A. and H.A. Lapham (2010) Assessing the reliability of criteria used to identify post cranial bones in sheep, *ovis* and goats *Capra.* Journal of Archaeological Science 37(11), 2887-2905.
